# Incidence of distal radius fracture surgery in Finns aged 50 years or more between 1998 and 2016 – too many patients are yet operated on?

**DOI:** 10.1186/s12891-018-1983-0

**Published:** 2018-03-02

**Authors:** Teemu P. Hevonkorpi, Antti P. Launonen, Tuomas T. Huttunen, Pekka Kannus, Seppo Niemi, Ville M. Mattila

**Affiliations:** 1 Department of Orthopedics and Trauma Surgery, Tampere University Hospital, Tampere, Finland; 2 Department of Anesthesia, Tampere University Hospital, Tampere, Finland; 3 School of Medicine, University of Tampere, Tampere, Finland; 4 Injury & Osteoporosis Research Center, UKK Institute for Health Promotion Research, Tampere, Finland; 5 Department of Clinical Science, Intervention and Technology, Karolinska Institutet, Division of Orthopedics and Biotechnology, Karolinska Institutet and Department of Orthopedics at Karolinska University Hospital, Karolinska Institutet, Solna and Huddinge, Sweden

**Keywords:** Distal radius, Epidemiology, Surgical treatment, Volar plating, Forearm fractures

## Abstract

**Background:**

Although optimal treatment of distal radius fractures is controversial, surgery has gained popularity. The purpose of this study was to evaluate recent trends in the surgical treatment of distal radius fractures in Finns aged 50 years or more.

**Methods:**

A nationwide hospital discharge register-based study was conducted among all patients 50 years of age or older who had a surgically treated distal radius fracture in Finland between 1998 and 2016. The number and rate of different surgical procedures were calculated per 100,000 person-years.

**Results:**

Altogether 21,965 surgically treated distal radius fractures were identified. During the study period the rate of percutaneous pinning and external fixation diminished while the rate of plate fixation significantly increased. The rate of operative treatment increased continually from 1998 to 2008 whereupon the peak of the incidence was achieved. After 2008, the rate of operative treatment of distal radius fracture remained quite constant, ranging between 61.1 and 67.8 per 100,000 person-years.

**Conclusions:**

Plate fixation has almost completely replaced both external fixation and percutaneous pinning in the surgical treatment of distal radius fractures in Finland. Despite growing evidence for less invasive treatment options in elderly patients, operative treatment of distal radius fracture is still rather popular today.

## Background

Distal radius fracture (DRF) is the most common fall-related fracture [1, 2]. Its incidence peaks in children less than 15 years of age and in adults after the age of 50 [2]. Women have a greater risk of sustaining a distal radius fracture than men. In a Swedish population-based study, the incidence of DRF was 390 per 100,000 person-years in women and 120 per 100,000 person-years in men, giving a female to male ratio of 3.3:1 [3]. The overall incidence of DRFs in adults varies between 100 to 300 per 100,000 person-years [2–4]. Concerning secular trends, recent studies from Sweden, the Netherlands and Austria show that the incidence of DRFs has stabilized [5–8].

Even though non-operative treatment is still the most common treatment option for distal radius fractures, many investigators have reported about increase in the surgical activity [7, 9, 10]. In a Medicare patient cohort in the U.S.A., Chung and co-workers observed a trend of a decreasing rate of non-operative treatment. Coincidental with the change, the rate of open reduction and internal fixation increased and the rate of percutaneous approaches decreased [10]. These changes have been later confirmed by national studies in Finland (from 1998 to 2008) and Sweden (from 2004 to 2010) [7, 9]. To the best of our knowledge, no national level epidemiological data on operative treatment of DRFs has been reported thereafter.

The trend towards open reduction and internal fixation (ORIF), mainly by volar locked plating, occurred internationally despite a lack of strong evidence to support its use. Recent high quality studies have shown that volar locked plating does not yield superior results over other surgical techniques after one-year follow-up [11–18]. In some studies, volar locked plating has been found to have advantages in comparison to external fixation and percutaneous pinning in the early rehabilitation period [13, 15, 17]. Long term results show, however, no difference between the methods [16, 18]. In addition, in the older age groups, non-surgical treatment seems to result in as good functional outcome as surgical treatment, however, with a lower rate of complications [11, 19].

The purpose of this study was to investigate recent trends in the incidence of DRF surgery in Finns aged 50 years or more and to assess to what extent these trends reflect current best evidence. We also examined the incidences of various types of surgical treatments performed for DRFs in patients aged 50 years or more.

## Methods

To study the changes in the rates of different surgical techniques of the distal radius fractures over time we studied all persons 50 years of age and older in Finland between the 1^st^ of January 1998 and 31^st^ of December 2016. The distal radius fracture surgery data was obtained from the Finnish National Hospital Discharge Register (NHDR), which is an electronic hospital data registry containing variables such as personal identification number, sex, domicile, external cause of injury, diagnoses, and all surgical procedures performed during the hospital stay. The NHDR is mandatory for all hospitals in Finland including all private and public hospitals. The NHDR has been widely used in epidemiological research and the quality of the register is reportedly excellent [20, 21]. As a register-based blinded analysis, the study did not have identifiable individual participants and thus neither informed consent nor ethics approval was needed.

All Finnish adults aged 50 or older who underwent a surgical procedure for a distal radius fracture (ICD-10 code S52.5 or 52.6) were included. To analyse the rates of different surgical techniques the procedures were categorised into three groups according to the Nomesco (Nordic Medico-Statistical Committee) procedural classification: percutaneous pinning (procedural codes NCJ64 and NDJ64), external fixation (procedural codes NCJ70 and NDJ70), and plate fixation (NCJ62 and NDJ62). The NHDR does not include laterality of the fracture and thus in this study we included only the first distal radius fracture operation of each patient.

The main outcome variable was the number of patients who had a certain surgical procedure (pinning, external fixation or plate fixation) for a distal radius fracture. To calculate the population-based rates of surgical treatment, the annual population statistics were obtained from the Official Statistics of Finland [22], a statutory, computer-based population register of the country. In 1998, there were 5.15 million inhabitants in Finland of which 1.67 million (32,4%) were aged 50 years or more. The population structure in Finland has changed towards older so that in 2016 there were 5.50 million inhabitants of which 2.25 million (40,9%) were at least 50 years old. As the distal radius fractures (per 100,000 person-years) were based on the entire 50-year-old or older population of Finland between 1998 and 2016, no statistical probability estimation methods (intrinsically needed in cohort-based estimations) were used. This was in full accordance with our previous nationwide studies [9, 23].

## Results

**Fig. 1:**
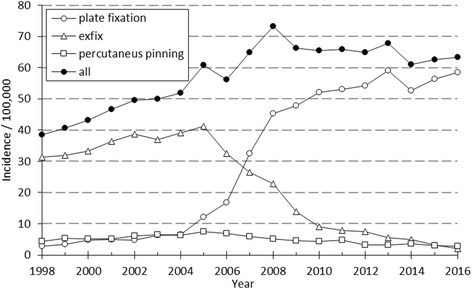
The incidence of surgical techniques used in the treatment of DRF in 50-year-old or older Finns between 1998 and 2016

A total of 21,965 DRFs in patients aged 50 years or more were treated surgically in Finland between 1998 and 2016. There were 3,916 (17.8 %) men and 18,049 (82.2 %) women. Open reduction with plate fixation was the most common technique in the operative treatment of distal radius fractures (*n*=12,108, 55.1%), followed by external fixation (*n*=8,020, 36.5%) and percutaneous pinning (*n*=1,837, 8.4%) (Fig. 1).

**Fig. 2:**
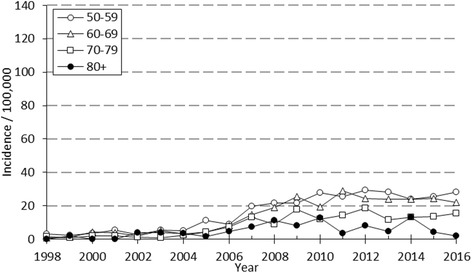
The annual incidence of plate fixation by age in 50-year-old or older Finnish men between 1998 and 2016

**Fig. 3:**
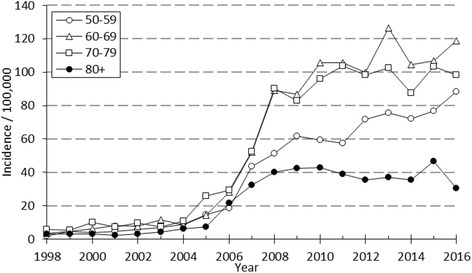
The annual incidence of plate fixation by age in 50-year-old or older Finnish women between 1998 and 2016

A marked change in the type of surgical fixation occurred during the study period as external fixation was replaced by open reduction and plate fixation as the most common procedure. After a moderate increase in the early 2000s there was a nearly six-fold increase in the incidence of plate fixation between 2004 and 2008, after which the rate of increase started to slow down. The incidence of operative treatment in women altered between 58.3 to 112.4 per 100,000 person-years being considerably higher than in men (13.6 to 28.5) during the study period. In men the rate of plating increased quite similarly in all age groups (Fig. 2) whereas in older women a striking increase in plating occurred after 2004 (Fig. 3). The peak of the incidence of ORIF occurred in 2013. Between 2013-2014 the incidence of plate fixations declined from 59.1 to 52.7 per 100,000 person-years. The decline was observed in women of all age groups, whereas in men the decline was observed only in patients aged 50 to 59. After 2014, the incidence of ORIF has increased moderately but continually, yet remaining below the peak incidence of 2013. Coincidental with the overall increase in the rate of plate fixation, a decrease in the rate of the two other surgical techniques, mainly external fixation, was observed.

**Fig. 4:**
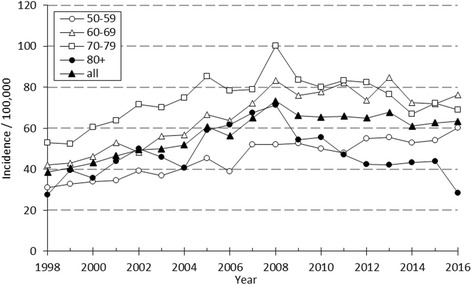
The annual incidence of surgical treatment of DRF by age in 50-year-old or older Finns between 1998 and 2016

The overall activity of operative treatment of DRFs reached its peak in 2008 (73.3 per 100.000 person-years). After that the incidence of external fixation has diminished year by year (from 22.9 in 2008 to 2.1 in 2016), thus stabilizing the increase in the use of ORIF. Between 2009 and 2016 the overall incidence of operative treatment of DRFs has remained rather stable, varying between 61.1 and 67.8 (Fig. 4).

## Discussion

The main finding of the present study was that despite emerging evidence supporting non-operative treatment methods of distal radius fractures in elderly patients, the operative activity of this common injury has remained notably high in older adults.

The increase in operative activity seemed to arise primarily from the increased activity of ORIF and simultaneous decrease in percutaneous fixation and especially external fixation. The change occurred quickly and without clear support of scientific evidence [11, 19]. Interestingly, the increase in surgical activity seemed to result mainly from the increased rate of surgical treatment of older women. In men the changes were more moderate. Incidence ratio of surgical treatment between women and men varied from 3.2:1 to 4.6:1 during the study period. Assuming that incidence ratio of the DRF has remained in approximately 3.3:1 in Northern Countries [3], women are operated to a slightly greater extent.

It has been previously reported that there has been a significant increase in operative activity, especially in volar locked plating, in the treatment of distal radius fracture [5, 7, 9, 10]. However, in our study we noticed the rate of operative treatment to stabilize after 2008. Nevertheless, despite the stabilization of the overall incidence of operative treatment, the number of DRF operations in older men and women has remained notably high. Even though volar locked plate fixation may result in better anatomical reduction compared to percutaneous techniques or non-operative treatment, we may anticipate that anatomical restoration does not automatically yield good functional outcome in elderly people [15, 17].

In a previous study, Koval and co-workers reported of a decreasing trend of percutaneous techniques in the treatment of distal radius fractures among younger orthopaedic surgeons in the United States [24]. In a selected Medicare patient cohort, Chung et. al. observed a decreasing trend of closed treatment and an increase in the surgical management of distal radius fractures. They also noted a trend towards ORIF [10]. Similar findings have been reported from Finland, Sweden and the Netherlands [5, 7, 9]. In this study, we also noticed this significant increase in incidence of operative treatment of DRFs (from 38.5 per 100,000 person-years in 1998 to 63.4 per 100,000 person-years in 2016). At the same time, it seems that the total incidence of distal radius fracture has stabilized or even decreased, according to studies by de Putter et al., Dimai et al. and Wilcke and colleagues [5, 7, 8]. Put together, these findings suggest that nowadays orthopaedic surgeons lean towards surgery in the treatment of distal radius fractures. This is of interest since there is no high-quality scientific evidence that warrants increased surgical activity in the treatment of elderly people’s distal radius factures [11, 19]. In fact, there is one randomized controlled trial supporting non-operative treatment of the distal radius fracture in the older age groups [11].

Unfortunately, the NHDR does not include laterality of the fracture and thus in this study we included only the first distal radius fracture operation of each patient. It is possible that same patient has had a contralateral distal radius fracture later on which has also been treated operatively. However, we consider that cases like this are infrequent and are unlikely to affect the results and conclusions of our study. In addition, nationwide incidence of distal radius fractures in Finland is not known since only patients treated operatively or hospitalised due to distal radius fracture are included in the NHDR. However, a previously published population-based study from Sweden showed that the incidence of distal radius fractures has not changed markedly during recent years [7]. Thus, we propose that the more pronounced incidence changes observed in the present study are unlikely to be explained by variations in DRF incidence alone. Smaller fluctuations in DRF surgery incidence, such as the 2008 peak or the decline after 2013, may well be related to the number of DRFs sustained by the population.

## Conclusions

In Finland plate fixation has almost completely replaced both external fixation and percutaneous pinning in the surgical treatment of distal radius fractures. The observed change, which has occurred especially in the older age groups, is not in line with the best available evidence on optimal treatment of distal radius fractures. As various surgical treatment methods and conservative treatment produce rather similar functional results, we should be critical in the use of volar locked plate fixation in the treatment of older adults’ DRFs. According to our results, current Finnish practice patterns in treating DRFs do not reflect the best available and most recent evidence. This should prompt a revision of the current Finnish Clinical Practice Guidelines on the treatment of DRFs.

## Abbreviations

DRF: Distal radius fracture
ICD: International Classification of Diseases
NHDR: National Hospital Discharge Register
Nomesco: Nordic Medico-Statistical Committee
ORIF: Open reduction and internal fixation
